# Supplementary material for: Testosterone-induced metabolic changes in seminal vesicle epithelium modify seminal plasma components with potential to improve sperm motility
Source: eLife. 2025 Dec 18;13:RP95541. doi: 10.7554/eLife.95541 (PMC12714332; doi:10.7554/eLife.95541)
Supplement: Figure 5—figure supplement 1—source data 2. [file elife-95541-fig5-figsupp1-data2.pdf]

Figure 5 figure supplement 1A

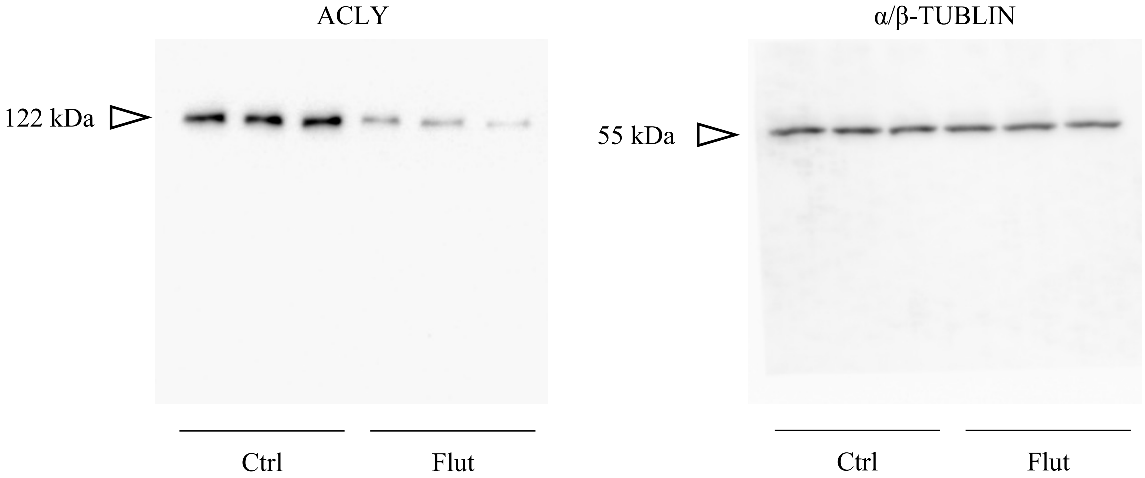

Figure 5—figure supplement 1—source data 2. PDF file containing original western blots for Figure 5 figure supplement 1A, indicating the relevant bands and treatments. Representative Western blot images of ACLY and α-tubulin in three sets of seminal vesicle collected from 50 mg/kg flutamide subcutaneously for 7 days (Flut) or vehicle treated mice (Ctrl).
