# Supplementary figures and images for: Testosterone-induced metabolic changes in seminal vesicle epithelium modify seminal plasma components with potential to improve sperm motility
Source: eLife. 2025 Dec 18;13:RP95541. doi: 10.7554/eLife.95541 (PMC12714332; doi:10.7554/eLife.95541)

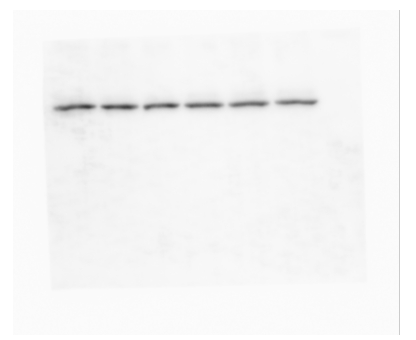

Supplement: Figure 5—figure supplement 1—source data 3. [file elife-95541-fig5-figsupp1-data3.zip › Figure 5 S1 Tublin.tiff]

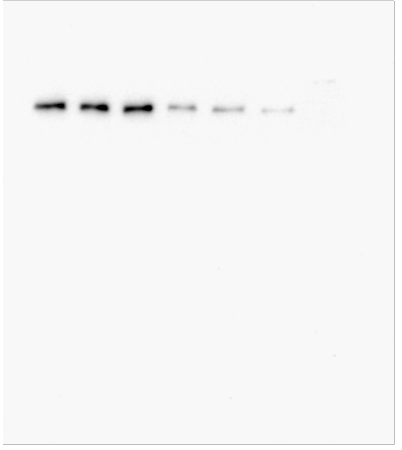

Supplement: Figure 5—figure supplement 1—source data 3. [file elife-95541-fig5-figsupp1-data3.zip › Figure 5 S1 ACLY.tiff]

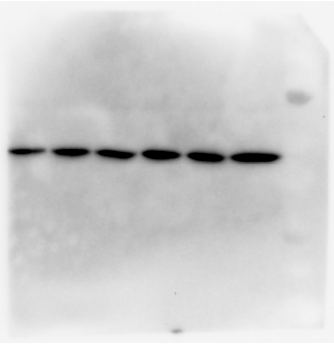

Supplement: Figure 6—source data 3. [file elife-95541-fig6-data3.zip › Figure 6 Tublin.tiff]

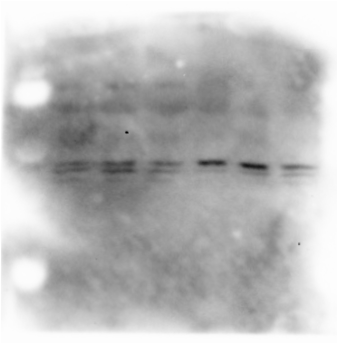

Supplement: Figure 6—source data 3. [file elife-95541-fig6-data3.zip › Figure 6 GLUT4.tiff]

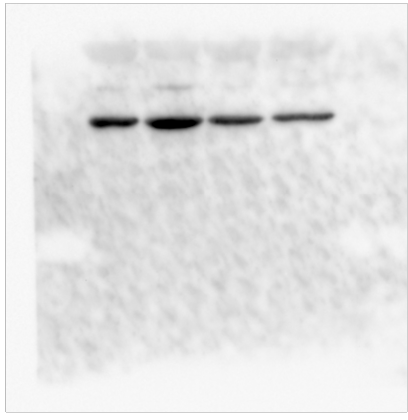

Supplement: Figure 7—source data 4. [file elife-95541-fig7-data4.zip › Figure 7C Tublin.tiff]

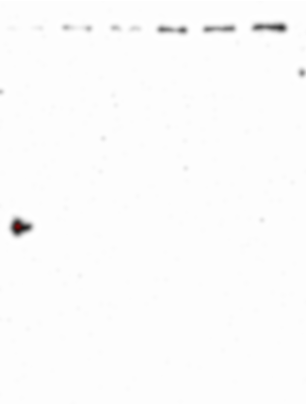

Supplement: Figure 7—source data 4. [file elife-95541-fig7-data4.zip › Figure 7A ACLY.tiff]

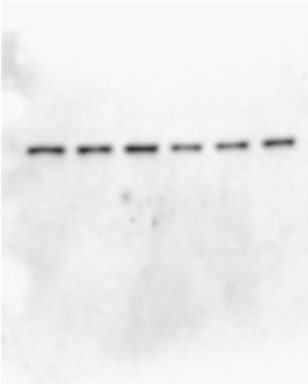

Supplement: Figure 7—source data 4. [file elife-95541-fig7-data4.zip › Figure 7A Tublin.tiff]

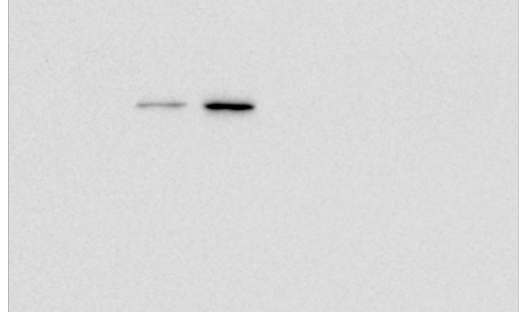

Supplement: Figure 7—source data 4. [file elife-95541-fig7-data4.zip › Figure 7C ACLY.tiff]
