# Supplementary material for: Testosterone-induced metabolic changes in seminal vesicle epithelium modify seminal plasma components with potential to improve sperm motility
Source: eLife. 2025 Dec 18;13:RP95541. doi: 10.7554/eLife.95541 (PMC12714332; doi:10.7554/eLife.95541)
Supplement: Figure 6—source data 2. [file elife-95541-fig6-data2.pdf]

Fig6D

GLUT4

50 kDa ▻

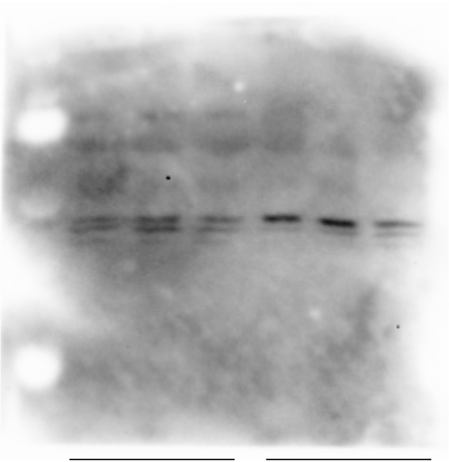

Ctrl Testo

α/β-TUBLIN

55 kDa ▻

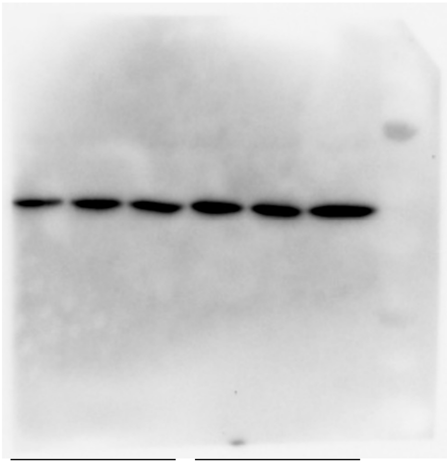

Ctrl Testo

Figure 6—source data 2. PDF file containing original western blots for Figure 6D, indicating the relevant bands and treatments. Western blot images of GLUT4 and α/β-tubulin in three sets of seminal vesicle epithelial cells cultured with 100 ng/mL testosterone (Testo) or in vehicle (Ctrl) for 7 days.
