# Supplementary material for: Testosterone-induced metabolic changes in seminal vesicle epithelium modify seminal plasma components with potential to improve sperm motility
Source: eLife. 2025 Dec 18;13:RP95541. doi: 10.7554/eLife.95541 (PMC12714332; doi:10.7554/eLife.95541)
Supplement: Figure 6—figure supplement 2—source data 2. [file elife-95541-fig6-figsupp2-data2.pdf]

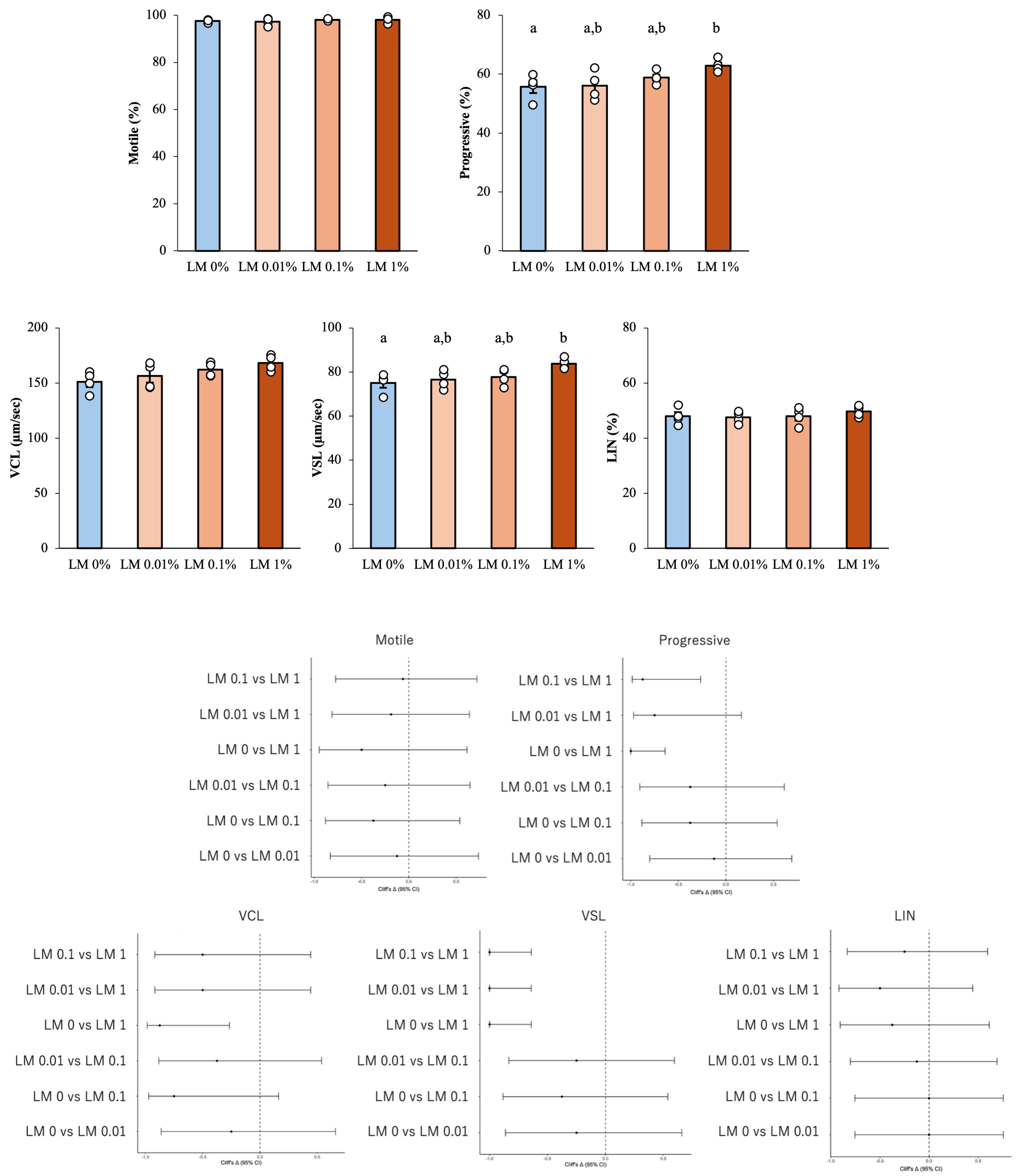

Results of reanalysis of Figure 6—figure supplement 2 using nonparametric tests and effect sizes with 95% confidence intervals Upper part; Differences between groups were assessed by Kruskal–Wallis test, differences among values were analyzed by Dunn's post-hoc comparisons (Bonferroni corrected) for multiple comparisons. Different letters represent significantly different groups. Lower part; The effect sizes with 95 % confidence intervals. For example, Cliff's Δ = -1 (95% CI ~ -0.6) in VSL's "LM 0 vs LM1" means that LM 1% values exceed LM 0 %values in all pairs.
