# Supplementary material for: Testosterone-induced metabolic changes in seminal vesicle epithelium modify seminal plasma components with potential to improve sperm motility
Source: eLife. 2025 Dec 18;13:RP95541. doi: 10.7554/eLife.95541 (PMC12714332; doi:10.7554/eLife.95541)
Supplement: Figure 7—source data 3. [file elife-95541-fig7-data3.pdf]

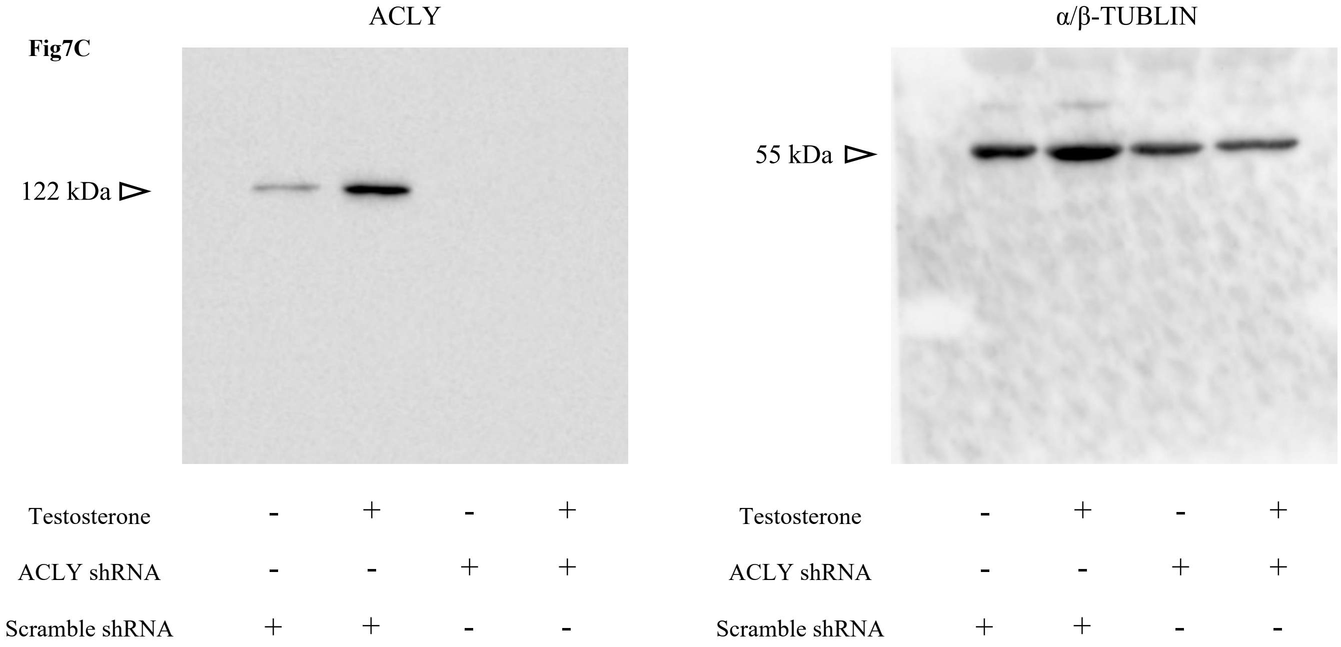

Figure 7—source data 3. PDF file containing original western blots for Figure 7C, indicating the relevant bands and treatments. shRNA knockdown experiments of ACLY in seminal vesicle epithelial cells. ACLY protein levels in scrambled shRNA or ACLY shRNA-transfected seminal vesicle epithelial cells cultured with or without 100 ng/mL testosterone were determined by Western blot.
