# Supplementary material for: Testosterone-induced metabolic changes in seminal vesicle epithelium modify seminal plasma components with potential to improve sperm motility
Source: eLife. 2025 Dec 18;13:RP95541. doi: 10.7554/eLife.95541 (PMC12714332; doi:10.7554/eLife.95541)
Supplement: Supplementary file 1. [file elife-95541-supp1.docx]

| **Supplementary file 1.** Primer sequences used for quantitative real-time PCR. | | |
| --- | --- | --- |
| Gene | Forward primer (5’→3’) | Reverse primer (5’→3’) |
| *Acc* | GCCTCTTCCTGACAAACGAG | TGACTGCCGAAACATCTCTG |
| *Acly* | AGGTCTCTCTGCAGCCATGT | AAGCTTTCCTCGACGTTTGA |
| *Aco2* | CTAGCCTCAGCCCAGTGAAC | CCTTCAGCCACCAGTGAAAT |
| *Cs* | CAGATGCCCACAGAGGAACA | AGCTTGGCAATGAGGTCCAT |
| *ElovI6* | CCCGAACTAGGTGACACGAT | CCGCAAGGCGTAGTAAGAGT |
| *Fasn* | AGCACACATCCTAGGCATCC | TGTCGTGTCAGTAGCCGAGT |
| *Fh* | AGCAATGCATATTGCTGCTG | CGCATACTGGACTTGCTGAA |
| *Slc2a1* | AAACATGGAACCACCGCTAC | AGGCCAACAGGTTCATCATC |
| *Slc2a2* | GCCTGTGTATGCAACCATTG | TGGCCCAATCTCAAAGAAAC |
| *Slc2a3* | TGTCACAGGAGAAGCAGGTG | GCTCCAATCGTGGCATAGAT |
| *Slc2a4* | ACTCTTGCCACACAGGCTCT | AATGGAGACTGATGCGCTCT |
| *Hk1* | TATCGGTCCAGCACGTATGC | ACATCGTGACCCACACAGTC |
| *Hk2* | GGGTAGCCACGGAGTACAAA | TGGATTGAAAGCCAACTTCC |
| *Hmgcr* | TGGAGATCATGTGCTGCTTC | GCGACTATGAGCGTGAACAA |
| *Idh2* | CCGTCTTCAGAGAGCCAATC | GAAATGGACTCGTCGGTGTT |
| *Mdh2* | GCTTTGTCTTCTCCCTCGTG | CAAAGTCCTCGCCTTTCTTG |
| *Nd1* | CAGGATGAGCCTCAAACTCC | CCGGTTTGTTTCTGCTAGGG |
| *Nd6* | GGTTGGTTGTCTTGGGTTAGC | TAGATCCCCAAGTCTCTGGA |
| *Ogdh* | ACATGGCACAGTGCATCATT | ACATCTGCAGAAACCGCTCT |
| *Pdha* | GGGGACGTCTGTTGAGAGAG | TGTGTCCATGGTAGCGGTAA |
| *Sdhb* | ACTGGTGGAACGGAGACAAG | GTTAAGCCAATGCTCGCTTC |
| *Suclg2* | CTTTGGTGGGATCGTCAACT | AACAGCTTTCTTGGCTGCAT |
| *L19* | GGCATAGGGAAGAGGAAGG | GGATGTGCTCCATGAGGATGC |
